# Barriers and facilitators of implementing electronic monitors to improve adherence and health outcomes in tuberculosis patients: protocol for a systematic review based on the Consolidated Framework for Implementation Research

**DOI:** 10.1186/s12961-023-01054-x

**Published:** 2023-11-01

**Authors:** Wenhui Li, Min Su, Weile Zhang, Xiaojing Fan, Renzhong Li, Yulong Gao, Xiaolin Wei

**Affiliations:** 1 School of Public Administration, Inner Mongolia University, Hohhot, China; 2 School of Public Policy and Administration, Xi’an Jiaotong University, Xi’an, China; 3 Chinese Center for Disease Control and Prevention, Beijing, China; 4 Inner Mongolia Center for Disease Control and Prevention, Hohhot, China; 5 Dalla Lana School of Public Health, University of Toronto, Toronto, Canada

**Keywords:** Tuberculosis, Electronic monitors, Implementation science, Barriers and facilitators

## Abstract

**Background:**

Tuberculosis (TB) has been regarded as ‘a relentless scourge’, increasing morbidity and mortality and burdening vulnerable populations. Poor adherence to TB treatment and ineffective traditional interventions hinders TB control. A novel TB approach called ‘electronic monitors’, equipping medication boxes with daily audio or visual reminders for electronically monitoring medication intake, seems promising in improving adherence and health outcomes and overcoming the weaknesses of traditional interventions. However, no review has systematically examined and synthesized the influencing factors of implementing electronic monitors. Implementation research offers the means to analyse the influencing factors of the implementation and its process, fitting well with the aim of this review. Therefore, the widely recognized Consolidated Framework for Implementation Research (CFIR), which offers a common taxonomy for evaluating intervention implementation, will be adopted to systematically identify barriers and facilitators of the electronic monitors for improving adherence and health outcomes in patients with TB.

**Methods and analysis:**

The systematic review will follow the Preferred Reporting Items for Systematic Reviews and Meta-Analyses (PRISMA) guidelines. Literature research will be conducted in five electronic databases (Ovid MEDLINE, CINAHL, EMBASE, Cochrane Library and Web of Science) to identify the barriers and facilitators of implementing electronic monitors in patients with TB. The CFIR will be used as a guide for categorizing and synthesizing the barriers and facilitators. Study screening, data extraction, quality appraisal and data analysis will be conducted by two independent reviewers. The use of additional reviewers will solve any disagreements between the two reviewers.

**Discussion:**

Given the increased prominence of TB epidemiology and the adherence problem of electronic monitors, there is a solid rationale for synthesizing the existing studies via the CFIR. The findings and conclusion of this review will lay bare the achievements and effectiveness of implementing electronic monitors, as well as the attendant gaps and limitations. Further strategies for facilitating the implementation of electronic monitors will also be explored. This review will be of essential significance for research and practice, supporting future academic research initiatives centred on patients with TB and aiding electronic monitor design in lowering the morbidity and mortality associated with TB disease.

*Trial registration number:* PROSPERO: CRD42023395747.

**Supplementary Information:**

The online version contains supplementary material available at 10.1186/s12961-023-01054-x.

## Introduction

Tuberculosis (TB) is an infectious respiratory disease caused by *Mycobacterium tuberculosis* [1] that has plagued hundreds of millions of people over the past 200 years. It has been called a ‘a relentless scourge’ [2]. TB remains a global concern, with considerably increasing morbidity and mortality, and a heavy burden for vulnerable populations. In 2021, the WHO estimated that about 10.6 million people were suffering from TB globally [3]; about 1.6 million people died from it that year [4]. TB-associated high morbidity and mortality were more likely to be seen in vulnerable populations in low- and middle-income countries [4, 5]. Therefore, to lower the global burden of TB, the WHO and the United Nations (UN)introduced the WHO End TB Strategy (2016–2035), aiming for a 50% reduction of incidence rate by 2025 [6] and making Sustainable Development Goal 3 stopping the spread of TB by 2030 [7]. However, despite these efforts, maintaining adherence to TB treatment continues to pose a significant challenge, jeopardizing the wellbeing of numerous patients and impeding progress in TB prevention and control [8, 9]. Poor adherence to TB treatment hampers various adverse consequences, including prolonged disease infectiousness, drug resistance, increased risk of relapse, and even death [10, 11]. Moreover, given the setbacks of the coronavirus disease 2019 (COVID-19) pandemic, it has been projected that this will need more effort to be put into action worldwide, particularly among the vulnerable. Time and effort must be invested in intervention measures [12] aimed at realizing long-term medication treatment and promoting the adherence and health outcomes of existing patients with TB.

A novel approach for improving the adherence of patients called ‘electronic monitors’ that equipped medication boxes with daily audio or visual reminders for electronic medication intake monitoring [13] seems to be promising as an intervention. Medication event monitoring systems (MEMS), also known as event monitoring devices for medication support, have been recommended by the WHO [13]. They have been applied extensively in primary medical institutions [14] and are considered the tool with most potential hopeful for TB treatment adherence improvement. An EMM makes it convenient and flexible for patients with TB to take their medication, as it gives instructions and reminders for dosing and refill, assists prescription follow-up, allows health providers to gather and utilize patient-centred histories of medication dosing data for further consultation and therapy, and helps avoid risky interrupted adherence and ensure a timely intervention [13]. Moreover, electronic medication monitoring (EMM) boxes, one of the categories of EMM, combine automated electronic devices with medication containers. They can be connected with smartphones to remind patients with TB regularly, in an audible and visual manner, to take their medication. They also inform caregivers instantly of medication usage behaviour, so swift action can be taken in case medication boxes remain unopened [13, 15, 16]. Such electronic monitors overcome the shortcomings of traditional adherence interventions [e.g. directly observed treatment, short-course (DOTS), video observed treatment (VOT) and short messaging service (SMS)] [13, 17–19], offering more details and equipped reminders for patients. For instance, DOTS fails to address all categories of tuberculosis disease, inadequately reduces TB transmission, and inconveniences patients and healthcare providers [20]. The feasibility and availability of VOT are restricted by requirements, particularly for live VOT, necessitating further evaluation across diverse populations and settings [13]. SMS interventions cannot verify medication authenticity, supervise medication dosage, substitute in-person healthcare encounters via messages texting, or demonstrate superior efficiency compared with DOTS and VOT, and issues with cellular reception and compatibility hinder access in rural areas without stable general packet radio service (GPRS) coverage and signals [13, 15, 21]. As mentioned earlier, the outstanding strengths of electronic monitors lie in remote live monitoring with flexibility and availability, resource-saving convenience and patient-centred medication history supervision and follow-up. Furthermore, patients with TB encountered difficulties adhering to the traditional treatment interventions, and efforts to improve adherence proved ineffective in addressing this issue [3, 8, 22–24]. It follows that this new improvement in digital health technology, proven to reduce TB mortality and morbidity, will facilitate acceptance by health consumers and providers; more importantly, it advances adherence by 45% [25]. The goal and strategy of the WHO could be achieved by the advisable and appropriate implementation of electronic monitor intervention, which seems feasible.

Different pieces of research have studied the efficiency and effectiveness of implementing electronic monitors to improve adherence and health outcomes of patients with TB [24]. Most studies focus on the evaluation of electronic monitors’ effectiveness, evaluating the return on investment (ROI) of pilot implementation [26] compared with traditional intervention [27], analysing cost-effectiveness using the Markov analysis model [28], evaluating robustness [29] and qualitatively analysing acceptability [30]. Additionally, a great many types of innovative digital health technologies interact with electronic monitors, resulting in a diversity of monitors, such as web- and Android-based applications [31, 32], digital adherence technologies (DATs) with ingestible sensors for digital pill boxes [33, 34] and evriMED devices which require a subscriber identity module (SIM) card to be inserted [35]; all these can yield benefits for patients with TB. A scoping review also summarized the category, function and impact of digital monitor intervention [36]. Meanwhile, the majority of previous systematic reviews have focused on and compared the impact of several TB adherence and outcomes interventions [37, 38]. Although the factors of TB adherence and outcomes have been discerned [38], for example, EMM and synchronous VOT have better adherence and outcomes since they bolster efficiency and convenience and save resources for patients and healthcare providers, the factors affecting implementing electronic monitors have not been dealt with, and no studies have systematically reviewed and summarized the influencing factors of implementing the electronic monitor intervention process in patients with TB. To better analyse and understand how the determinants facilitate or undermine the implementing process of electronic monitors, the results and characteristics of various studies still need to be combined in one article.

Implementation research is regarded as a scientific study of methodologies and tactics that assists practitioners from evidence-based practice to routine adoption. It offers a perspective for analysing the influencing factors of implementation and its process, aiming to understand how the interventions work effectively within real-world settings as well as the reasons and contexts within the interventions [39]. It also allows for scaling up and optimizing the implementation of digital technologies related to TB in further research [40]. Theories and frameworks applied together with implementation research are more likely to function as a robust guide and basis for successful implementation, as well as allowing a novel perspective for exploring the determinants of intervention [41]. The complexity of planning, modifying, executing and maintaining an intervention has led to a pressing need for a theory- and framework-based implementation that gives both researchers and practitioners a concise, coherent, structured and comprehensive way of gathering effective contributing factors for the implementing process [42]. The Consolidated Framework for Implementation Research (CFIR) is a well-recognized and comprehensive ‘determinant framework’ that can be utilized to assess barriers and facilitators of implementing TB electronic monitors [43]. The CFIR, which draws upon 19 theories and frameworks, offers a common taxonomy for systematically evaluating intervention implementation and is particularly suitable for examining the effectiveness of implementation in the context of health service delivery [43, 44].

Given the above, this review will use framework-based implementation research to systematically examine, identify and synthesize the barriers and facilitators of implementing electronic monitors aiming to improve adherence and health outcomes in patients with TB. The three main objectives will be: (1) to identify the influencing factors of implementing electronic monitors in patients with tuberculosis; (2) to explore how barriers and facilitators make a difference in implementing electronic monitors according to framework-based implementation research; and (3) to analyse to what extent implementation science methods and theories are employed in the design, implementation, and assessment of electronic monitors in patients with TB for optimizing further implementation.

## Methods

### Study design

**Fig. 1:**
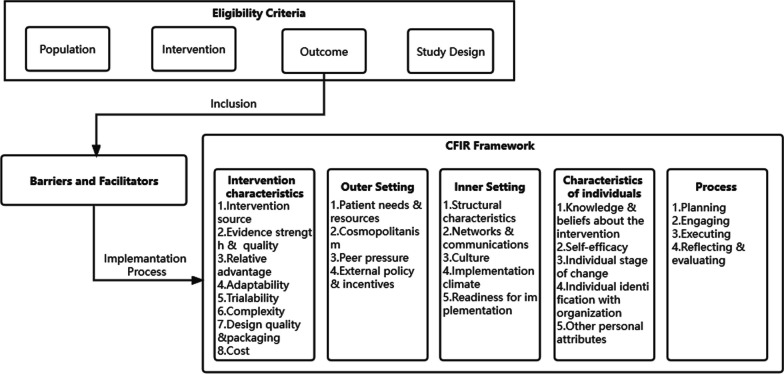
Theoretical model

The methods of the systematic review will follow the guidelines of the Preferred Reporting Items for Systematic Reviews and Meta-Analyses (PRISMA) [45], and the protocol adopts the PRISMA-P checklist [46] (see Additional file 1) as a guide. To assist the protocol development, a preliminary literature assessment will be conducted in the MEDLINE database via Ovid with lists of search terms as a pilot, and search terms will be added or deleted according to the search results skimmed and scanned by WL and WZ and double-checked by WL and MS to ensure accuracy. In this way, the search terms, the eligibility criteria for selection, the data extraction items and the analysis and synthesis frameworks and theories will be refined. The final protocol after adjustment and pilot, which has been registered in PROSPERO (CRD42023395747), is described in the following section. The theoretical model for this study is the combination of theoretical framework and eligibility criteria (see Fig. 1).

#### Theoretical framework

The conceptual framework that will be used to evaluate the influencing factors for the implementation of electronic monitors in patients with TB is the CFIR, which is regarded as one of the most influential conceptual frameworks in the field of implementation science [47]. It is composed of 5 domains (intervention characteristics, outer setting, inner setting, individual characteristics and process) and 39 sub-domains and serves to identify the barriers and facilitators of the implementation of electronic monitors: what works, where, for whom and why [43]. Furthermore, CFIR offers a comprehensive structure for combing and comparing the existing literature that fits well in programs with multiple levels and components [43], giving the reviewers a rigorous and systematic way to examine the determinants of implementing electronic monitors. CFIR is well suited to assessing the specific factors that facilitate and impede the electronic monitor implementation process, enabling researchers to evaluate these factors and their interrelationships.

### Eligibility criteria

The eligibility criteria for this research will include Population, Intervention, Outcomes, and Study design (PICOS) [48], while the Comparison (C) component will be excluded to expand the pool of available studies for the review. See Table 1 for comprehensive inclusion and exclusion criteria and additional criteria outside the PICOS framework.

**Table 1:** Inclusion and exclusion criteria

	Inclusion criteria	Exclusion criteria
Population (P)	• Patients diagnosed with pulmonary tuberculosis disease and using of electronic monitors	• Patients diagnosed with diseases other than TB or not using electronic monitors in their treatment
Intervention (I)	• Electronic monitors including an automated electronic device as a medication container or box	• Monitors with no automated electronic device as a medication container or box and without monitor function
Outcome (O)	• Factors that influence the implementation process of electronic monitors that aim to improve adherence and health outcomes of patients with TB	• No factors as categorized in the CFIR constructs that influence the implementation process of electronic monitors
Study design (S)	• Any study design expects those involving exclusion criteria	• Editorial and commentary articles, reviews and protocols studies which do not include intervention implementation or the affecting factors; assessment of guideline studies
Others	• No limits on publication year	• Literature not published in English
	• Includes human research	• Animal research

Population (P) denotes all patients who are diagnosed with pulmonary tuberculosis disease and use electronic monitors. Since the diagnostic criteria for TB have been undergoing change, no restriction will be applied to these. No age limitation will be applied for the patients either.

Intervention (I) denotes electronic monitors, including an automated electronic device as a medication container or box that aims to monitor patients with TB in taking their medication.

Outcome (O) denotes the factors that influence the implementation process of electronic monitors that aim to improve adherence and health outcomes of patients with TB. Barriers and facilitators will be categorized and measured by the CFIR framework.

Study design (S) denotes systematic and comprehensive design which takes all the literature into account.

Exclusion criteria for the population (P) denotes patients diagnosed with conditions other than TB or without electronic monitors as part of their treatment. Intervention (I) denotes monitors devoid of an automated electronic device serving as a medication container or box and lacking monitoring functionality. Outcome (O) denotes the absence of CFIR-categorized factors that influence the implementation of electronic monitors. Study design (S) denotes editorial articles, commentary, reviews and protocol studies that exclude intervention implementation or investigation of influencing factors, along with assessments of guideline studies.

### Search strategy

#### Search sources

Five critical electronic databases will used for literature search: MEDLINE via Ovid, CINAHL, EMBASE, Cochrane Library, and Web of Science. These are selected because they cover a specific area in health, medicine, nursing, biomedicine and allied health-related topics, enabling the identification of barriers and facilitators of implementing electronic monitors aimed at improving adherence and health outcomes in patients with TB. In addition, citation searches and reference list tracking will be carried out in Google Scholar and Google to retrieve further relevant information from critical studies. Grey literature searches of the first 20 pages Google Scholar and Google will screen for potentially useful studies to ensure the robustness of the search strategy.

#### Search terms

To ensure a robust and comprehensive search, the keywords will be adjusted after the pilot. Consequently, the keywords that will be searched in the electronic databases are ‘electronic monitors’ and ‘tuberculosis patients’. Medical subject headings (MeSH) terms, thesaurus terms, and free text searching will also be considered throughout the search to expand the screening scope, with a slight adjustment among different databases (see Additional file 2).

The following terms will be utilized in this protocol:

Tuberculosis (tuberculosis, TB, tuberculosis patients, pulmonary tuberculosis, lung tuberculosis).

Electronic monitor (electronic monitors, electronic pillboxes, electronic pill boxes, electronic medication boxes, digital pill boxes, digital pillboxes, digital medication boxes, smart pill boxes, smart pillboxes, SPs, smart medication boxes; medication monitors, medication monitor boxes, pill monitors, pill monitor boxes, electronic reminders, Internet reminders, technology reminders, digital reminders, technology interventions, digital interventions, electronic interventions, Mhealth, mobile health, Ehealth, electronic health, health technology, digital technology, digital health, digital health technology;

EMM, event monitoring device for medication support, EMM boxes, MEMS, medication event monitoring system;

health supporters, health devices, health applications, health websites, health software).

Boolean operators (AND, OR) will be applied across keywords to search and select the most comprehensive range of literature. The ‘AND’ operator will be used to combine different keywords, and the ‘OR’ operator will be used with similar terms or search terms that may have a less significant effect on expanding the search field.

#### Search limitation

No additional limitations will be imposed on the search for fear of missing out on critical abstracts and full-text publications with a time gap and to assist the completeness of the screening of the influencing factors of implementing electronic monitors in patients with TB. However, due to retrieval times varying between the databases, the starting time of the searches may be different. That said, the search language will be restricted: only English literature will be included in the search.

### Study selection

The study selection process will follow the PRISMA diagram, which is available on the PRISMA statement website [49]. Two independent reviewers (WL and WZ) will select studies according to the inclusion criteria and exclude literature according to the exclusion criteria. Following the PRISMA diagram will allow the two reviewers to minimize selection bias while searching occurs, from identification, screening and included stages, with the numbers of and reasons for included and excluded studies documented by electronic databases and other sources [49]. The screening process will also be recorded in the Excel worksheet and Mendeley Reference Manager.

Studies will first be screened via duplicate records checked by the two reviewers independently. After the duplication check, the screening process will start with the browsing of the titles in five databases and other sources to filter irrelevant articles (those who show none of the keywords in their title). The remaining articles will then be filtered, with their abstract searched for potential relevance. Those titles with possible significance but without a complete abstract or a vague statement in the abstract section will need to be checked in a subsequent review to determine whether they should be included. Meanwhile, the two reviewers will compare the results of the independently retrieved studies and put forward certain articles for further searching. In the following full-text screening, inclusion and exclusion criteria will need to be identified through browsing. Those qualified by the PICOS framework as discussing the correct target population, intervention types, study design, influencing factors, adherence and health outcomes will be included. However, the articles for which a complete text cannot be found will be excluded. By browsing and assessing the full-text articles of the remaining studies, articles that meet all the eligible inclusion criteria will be chosen as the final analysed and synthesized studies. The screening will be reported in a PRISMA diagram [49]. Any disagreement between the two reviewers throughout the screening process will be solved by discussion and consensus to make the study selection as objective as possible. XF, the third reviewer, will help solve disagreements by double checking the title, abstract and full text within the results of two independent reviewers.

### Quality assessment

Quality assessment for this review will be conducted via the Mixed Methods Appraisal Tool version 2018 (MMAT) [50]. The MMAT appraises all five categories of studies: qualitative and quantitative randomized controlled trials, quantitative non-randomized trials (cross-sectional, cohort and case–control studies), quantitative descriptive research and mixed methods studies [50]. Therefore, it will permit the reviewers to include all the determinants, adherence and health outcomes in patients with TB without being restricted by study type. MMAT works in two-part questions: part one serves for all five methodological study categories, and part two separates the studies according to five critical questions and requiring detailed interpretation. Each criterion is responded to with ‘Yes’, ‘No’, or ‘Can’t tell’ [50]. Both the methodology quality and risk of bias of the final included articles will be evaluated and assessed independently by WL and WZ according to the MMAT tool. Moreover, they will rank the quality of the selected articles as low, moderate or high on the basis of the MMAT criteria [51]. YG will act as third reviewer in these processes and double assess the studies’ quality to ensure credibility.

### Data extraction

Data will be extracted by browsing the whole text to identify information needed for the affecting factors, adherence and health outcomes of implementing electronic monitors in patients with tuberculosis. It starts from the abstract, introduction, method, results, discussion and conclusion sections of the included articles that qualified all the inclusion criteria. The whole process will be documented in a modified data extraction form followed by the guideline of the Cochrane Handbook [52], which provides a standard format for identifying homogeneity and heterogeneity among the selected studies and offers details for later analysing and synthesizing. The Cochrane Handbook will give WL and WZ a transparent and straightforward overview of the data extraction process [52] and will also be beneficial in reducing reporting bias. RL will be the third reviewer. After the piloting of the data extraction form, which aims to examine its generalization and effectiveness as used in this review, sections of critical findings in the extraction form will be replaced by the barriers and facilitators of implementing electronic monitors as well as adherence and health outcomes in patients with TB. Furthermore, CFIR constructs applied in five detailed categories domains analysing the determinants will also be considered and incorporated in this form, and all of the extracted determinants will fall into one of 39 sub-domains of CFIR. Similar to other studies that utilized CFIR in analysing the affecting factors related to TB [53–56], for example, questions in survey tools regarding TB and human immunodeficiency virus (HIV) in line with CFIR constructs when identifying ascertaining factors of tuberculosis preventive therapy [53], in this review, each CFIR construct will also be given definitions which are relevant and feasible to our context for information trustworthiness (see Additional File 3 for adjusted codebook within the CFIR). For example, attitudes regarding TB electronic monitors may fall within ‘knowledge and beliefs about the intervention’ constructs, while advantages of electronic monitors over DOT could fall within ‘relative advantage’ constructs. Given this, the finally customized form will contain the author and date, title, country, setting, participants, interventions, study design, health outcomes, adherence, barriers and facilitators as categorized in CFIR constructs. The data extraction form will be produced in an Excel sheet.

### Data analysis and synthesis

A narrative synthesis drawing on thorough reading will be conducted to summarize the selected studies’ general characteristics, determinants, adherence and health outcomes of implementing electronic monitors in patients with TB. The common barriers and facilitators will first be categorized by the five domains of the CFIR framework. Furthermore, the relationships of the determinants across and within the domains will be categorized, tabulated and examined. After identifying and categorizing the determinants, the analysis will prioritize exploring the impact of these determinants on implementing electronic monitors. It will also examine how to incorporate the identified barriers and facilitators into routine TB management, considering the perspective of the implementation science methods applied in these studies. The narrative data analysis process will be conducted by WL and WZ independently and in accordance with the four steps of the general framework of narrative synthesis [57]: constructing a theory for the intervention of how, why and for whom; developing an initial synthesis; examining the connections within and across the studies; and assessing its vigour [57]. The constructed theories will be mapped into the domains derived from the CFIR, and an initial synthesis will be regarding TB electronic monitors and the underlying implementation science theories of individual factors and the relationships among barriers and facilitators, which align with the process of achieving the main objectives in this review. Vigour assessment, cross-checked by reviewers, as well as adherence and health outcomes analysis, will also be synthesized accordingly. Narrative synthesis will assist reviewers and readers in interpreting and understanding the data and information extracted from the articles more accurately, systematically and comprehensively.

Additionally, deductive content analysis will be utilized to synthesize the qualitative data on barriers and facilitators, adherence and health outcomes of patients TB. Deductive content analysis is composed of three phases: preparation, organizing and reporting [58]. Affecting factors, adherence and outcomes from qualitative studies will first be identified in the preparation stage and then organized into the second stage. It applies a thematic framework to the process of data coding the qualitative data, starting with producing a preliminary code with a defined structure before reviewing [59]. Essential data will first be selected and then coded into categories using the CFIR framework, as initial coding facilitates a better integration of a large amount of literature for the reviewers [60]. In this review, the CFIR framework and codebook [61], as well as the codebook for influencing factors of implementing interventions to improve rational antibiotic use [62], will be used to guide the qualitative data coding in further grouped themes (see Additional File 3). In this way, the deductive data coding approach of synthesizing the influencing factors will be in accordance with the questions in the sub-domains of the CFIR framework. TB adherence and health outcomes will be assessed throughout the analysis based on patients’ transcripts. The research will also delve into how factors influenced the implementation of electronic monitors and the theories and methods of implementation science employed in the qualitative studies. This synthesis within the deductive content analysis will contribute to integrating influencing factors into daily TB practice, optimizing the implementation process, and ultimately improving adherence and health outcomes. Applying the deductive content analysis approach makes rich interpretive analysis possible [63]. Two reviewers will code the articles independently and compare the results with modifications until a consensus is reached. XW and MS will act as the additional reviewers here.

Furthermore, accomplishments and gaps in implementing electronic monitors to improve adherence and health outcomes in patients with TB will be identified and appropriate strategies for facilitating the implementation explored.

## Discussion

Taking into account the increasing urgency of TB epidemiology and the adherence problem of electronic monitors, there is a solid rationale for synthesizing the existing studies via an implementation science framework. This review will analyse the barriers and facilitators of implementing electronic monitors that aim to improve adherence and health outcomes for patients with TB across the CFIR framework domains.

The CFIR provides a thorough and structured approach to comprehending complex factors influencing intervention implementation. While the CFIR has been utilized in various healthcare contexts, its specific application to implementing electronic monitors in TB care remains relatively limited.

Previous studies employed the CFIR to identify and leverage barriers and motivators in TB control, such as disease texting, screening, triage, diagnosis, prescription, treatment, preventative and care improvement [53–56, 64–69]. Furthermore, it examined the challenges and enablers of implementing electronic monitors, other similar mobile health applications and electronic monitoring systems in diverse settings, including China, Uganda, North America and Africa, and South Africa [70–74]. These studies have illuminated the utility of the CFIR in exploring factors related to TB, electronic monitors and other digital health technologies and elucidating valuable insights into TB management and diverse healthcare settings.

Additionally, the CFIR has been effectively utilized in conjunction with electronic monitors and innovative adherence monitors to assess contextual deterrents in other diseases, such as HIV and asthma [75–79]. This extension of the CFIR beyond TB control across diverse health-related conditions demonstrates its versatility and applicability in a holistic understanding of implementation barriers and facilitators associated with similar electronic monitoring technologies.

In line with these insights, this review will contribute to the pioneering use of the CFIR in investigating the factors influencing the implementation of electronic monitors to enhance treatment adherence and improve health outcomes for patients with TB. The findings of this review will shed light on the accomplishments and challenges related to the implementation of electronic monitors, providing valuable insights for stakeholders and researchers to make necessary adaptations when implementing similar interventions. By comprehending the interconnectedness among these influencing factors, there will be an opportunity to optimize the implementation of electronic monitors, thus fostering the development of more effective ones.

### Strengths and limitations

This systematic review’s main strength is that it will be purposefully broad in electronic monitor issues in its treatment of patients with TB and is anticipated to cover a wide range of literature. This will allow the review to encompass all related and relevant information and enable its reviewers to capture and build a comprehensive overview of the current state of this research field. More importantly, this review will be the first to analyse the barriers and facilitators of implementing electronic monitors in patients with TB to improve adherence and health outcomes guided by a consolidated framework taken from implementation research.

The limitations of this review include that it will exclude studies not written in English; this may lead to selection bias and the omission of potentially significant articles written in other languages. Moreover, due to the relatively limited literature on electronic monitors, only ‘electronic monitors’ and ‘TB’ will be used as search terms, without ‘influencing factors’. The selection bias may also still exist despite a consensus being reached after the piloting of the search terms that the final search terms can cover the scope of literature.

Existing studies examined the application of the CFIR in both pre- and post-implementation stages. These studies have demonstrated the strengths of utilizing the CFIR in various types of research, including qualitative [54, 55, 65–67, 69, 71, 73, 75, 77, 79], quantitative [53], mixed methods [76] and case studies [72]. Qualitative studies have employed the CFIR as an interview design and guide and collection tool to explore the perspectives and engagement of healthcare system stakeholders concerning implementation readiness [54, 55, 65–67, 69, 71, 73, 75, 77, 79]. However, it should be noted that the success of information capture may vary across specific constructs [66].

The pre-implementation stage focusses on assessing the feasibility and acceptability of CFIR-driven planning. It helped identify factors that promote or undermine implementation success, guiding practical and programmatic implications. Additionally, it also allowed for the evaluation of scalability and generalization across cities and countries [55, 66, 68, 69, 73, 79].

Furthermore, the post-implementation stage aims to address potential challenges and optimize motivators to improve adherence and refine health workers’ behaviour change with better service. It investigated evidence-based approaches, from TB identification to follow-up treatment, to enhance health outcomes. Furthermore, it identified modifiable factors that could facilitate future scaling-up and dissemination efforts [53, 54, 65, 67, 71, 72, 75–77].

This review will incorporate various study types to fully leverage the CFIR framework’s benefits. By utilizing the CFIR framework, we aim to comprehensively characterize and anticipate the barriers and facilitators in introducing TB electronic monitors, both dependently and independently, in a specific setting and across different contextual locations. This framework will allow us to identify and overcome implementation challenges, generalize findings and optimize future implementations. Moreover, it will provide valuable insights into the factors that influence the successful implementation of TB electronic monitors, empowering us to offer well-informed recommendations for enhancing the implementation process.

### Relevance of the review

As specified in this protocol, the systematic review intends to explore, evaluate and synthesize the evidence for the barriers and facilitators of implementing electronic monitors in patients with TB. Consequently, an evidence basis will be provided in the review regarding the use of framework-based implementation research in studying electronic monitors aimed at improving adherence and health outcomes in patients with TB. The findings and conclusion will detail the achievements and effectiveness of implementing electronic monitors as well as gaps and limitations. Further strategies for facilitating the implementation of electronic monitors in this context will also be explored. Information provided in the review will be of essential significance for research and practice, supporting future academic research initiatives centred on patients with TB as well as aiding in the design of electronic monitors for lowering the morbidity and mortality associated with TB.

### Ethics and dissemination

**Fig. 2:**
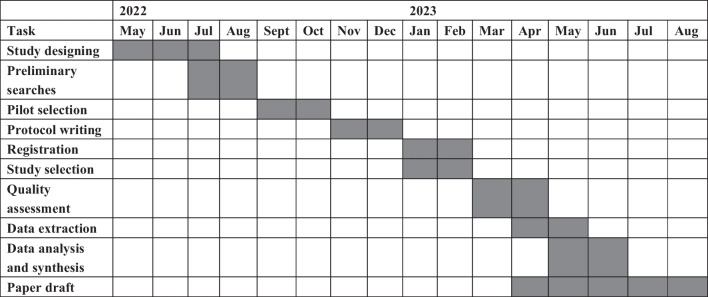
Timeline

Ethics approval is not required for this systematic review, as it intends to synthesize published literature. This study will span 16 months, encompassing various phases to ensure a thorough review process. The initial 3-month phase will focus on study design, while preliminary searches will be conducted simultaneously in July. Following that, a 2-month period will be allocated for pilot selection, protocol writing and the concurrent processes of registration and study selection. From March to April, the quality assessment will be performed to ensure the reliability and validity of the collected data. At the same time, data extraction will commence in April and continue into May, involving retrieving relevant data from the selected studies. Once data extraction is complete, the subsequent data analysis and synthesis phase will begin in May, extending until July. The collected data will be analysed, interpreted and synthesized during this phase to derive meaningful insights and draw conclusions. It is worth noting that the quality assessment, data extraction and data analysis and synthesis activities will overlap within the same month to ensure a cohesive and efficient research endeavour. The final phase of the study will involve the write-up process, spanning from April to August, where the study findings will be synthesized, and a comprehensive manuscript will be prepared (see Fig. 2 for more details). Ideally, the review findings will be submitted to peer-reviewed journals, presented at national and global conferences and shared with key stakeholders, including health service authorities, healthcare providers and general patients with tuberculosis.

### Supplementary Information

**Additional file 1.** PRISMA-P checklist.

**Additional file 2.** Search strategy.

## Data Availability

All data analysed in this study is included in this article.

## Abbreviations

TB: Tuberculosis
MEMS: Medication event monitoring systems
EMM: Event monitoring device for medication support
EMM boxes: Electronic medication boxes
DOTS: Directly observed treatment, short-course
VOT: Video observed treatment
SMS: Short messaging service
PRISMA: Preferred Reporting Items for Systematic Reviews and Meta-Analyses
CFIR: Consolidated Framework for Implementation Research
PICOS: Population, Intervention, Comparison, Outcomes and Study design
GPRS: General packet radio service
MeSH: Medical subject headings
MMAT: Mixed Methods Appraisal Tool version 2018
