# Supplementary material for: Barriers and facilitators of implementing electronic monitors to improve adherence and health outcomes in tuberculosis patients: protocol for a systematic review based on the Consolidated Framework for Implementation Research
Source: Health Res Policy Syst. 2023 Nov 1;21:115. doi: 10.1186/s12961-023-01054-x (PMC10621129; doi:10.1186/s12961-023-01054-x)
Supplement: Supplementary file 2 — Additional file 2. Search strategy. [file 12961_2023_1054_MOESM2_ESM.docx]

**Additional file 2**- Medline Search Strategy

**Tuberculosis (population)**

1 tuberculosis/ or tuberculosis. mp. or tuberculosis, pulmonary/ 251808

2 tuberculosis patient*.mp. 6895

3 exp Tuberculosis/ 204471

4 TB .mp. 57285

5 ((lung* or pulmonary*) adj3 (tuberculosis* or TB)) .mp. [mp=title, book title, abstract, original title, name of substance word, subject heading word, floating sub-heading word, keyword heading word, organism supplementary concept word, protocol supplementary concept word, rare disease supplementary concept word, unique identifier, synonyms] 85175

6 (tuberculosis* or lung tuberculosis* or pulmonary tuberculosis*) .mp. [mp=title, book title, abstract, original title, name of substance word, subject heading word, floating sub-heading word, keyword heading word, organism supplementary concept word, protocol supplementary concept word, rare disease supplementary concept word, unique identifier, synonyms] 251822

7 1 or 2 or 3 or 4 or 5 or 6 270025

**Electronic monitor(intervention)**

8 (electronic monitor* or electronic pillbox* or electronic pill box* or electronic medic* box* or digital pill box* or digital pillbox* or digital medic* box* or smart pill box* or smart pillbox* or SPs or smart medic* box*) .mp. [mp=title, book title, abstract, original title, name of substance word, subject heading word, floating sub-heading word, keyword heading word, organism supplementary concept word, protocol supplementary concept word, rare disease supplementary concept word, unique identifier, synonyms] 7219

9 (medic* monitor* or medic* monitor* box* or pill* monitor* or pill monitor* box* or electronic* reminder* or Internet reminder* or tech* reminder* or digital reminder* or tech* intervention* or digital intervention* or electronic* intervention* or Mhealth* or mobile* health* or Ehealth* or electronic health* or health tech* or digital tech* or digital* health or digital health tech*) .mp. [mp=title, book title, abstract, original title, name of substance word, subject heading word, floating sub-heading word, keyword heading word, organism supplementary concept word, protocol supplementary concept word, rare disease supplementary concept word, unique identifier, synonyms] 69617

10 (EMM* or event monitoring device for medication support* or EMM box* or MEMS*or medication event monitoring system*) .mp. [mp=title, book title, abstract, original title, name of substance word, subject heading word, floating sub-heading word, keyword heading word, organism supplementary concept word, protocol supplementary concept word, rare disease supplementary concept word, unique identifier, synonyms] 10083

11  (health* adj3 (supporter* or device* or app* or web* or software*)) [mp=title, book title, abstract, original title, name of substance word, subject heading word, floating sub-heading word, keyword heading word, organism supplementary concept word, protocol supplementary concept word, rare disease supplementary concept word, unique identifier, synonyms] 75908

12 8 or 9 or 10 or 11 157583

**All combined**

13 7 and 12 1326
